# Incidental Finding of an Undiagnosed Coarctation of the Aorta Causing Dilated Cardiomyopathy in an Adult

**DOI:** 10.1155/2017/6129073

**Published:** 2017-07-27

**Authors:** Abdalla Ibrahim, Zahir Satti, Ronan Curtin

**Affiliations:** Cork University Hospital, Wilton, Cork, Ireland

## Abstract

31-year-old male with no past medical history apart from high blood pressure noted by GP one week prior to admission presented with a three-week history of a flu-like illness and symptoms of heart failure with severe global left ventricular dilation and dysfunction on Transthoracic Echocardiography (TTE). Two weeks following admission he complained of left arm pain and CT upper limb confirmed embolic occlusion of the left brachial artery and incidental severe coarctation of the proximal descending aorta after the origin of the left subclavian artery. Follow-up TTE suggested the presence of coarctation of the aorta on a suprasternal view which was not performed at the time of his first TTE. His heart failure and blood pressure responded very well to medical therapy and he has been referred for surgical correction of his aortic coarctation.

## 1. Introduction

We present an extremely rare case of dilated cardiomyopathy as a presentation of aortic coarctation in an adult patient.

## 2. Case Presentation

**Figure 1:**
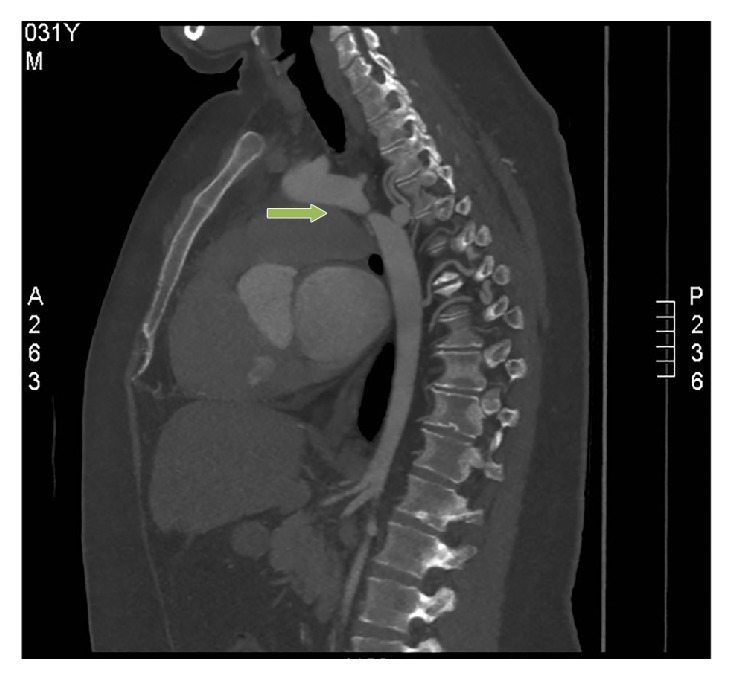
CT of left upper limb showing coarctation of the aorta at the isthmus.

**Figure 2:**
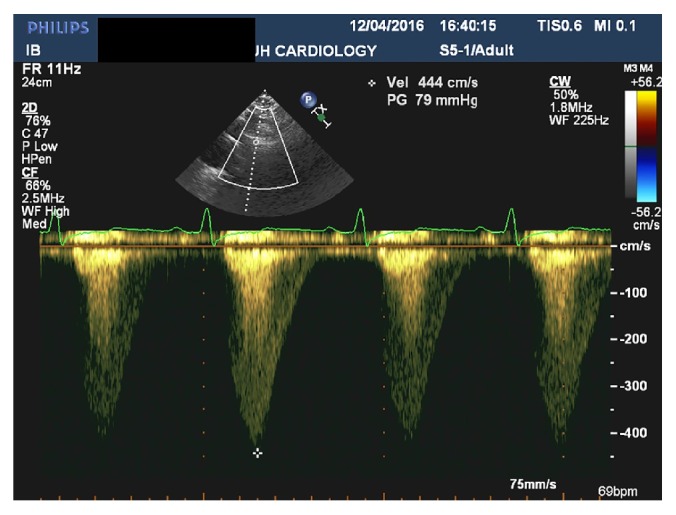
Suprasternal view of TTE with Doppler showing a peak gradient of 79 mmHg suggesting the presence of aortic coarctation.

31-year-old male presented with dyspnoea on exertion NYHA class II, orthopnoea, and lower limb oedema for three weeks. These symptoms were preceded by flu-like symptoms. He was also noted to be hypertensive by his GP a week prior to admission and was started on Ramipril. He has no other significant past medical history apart from a high Body Mass Index of 38. He consumed about 40 units of alcohol per week and he has a positive family history of ischaemic heart disease (father and two grandfathers had myocardial infarction at the age of 50). Examination revealed a blood pressure of 160/70 mmHg, heart rate of 110 beats per minute, oxygen saturation of 97% on room air, raised jugular venous pressure, bibasal crepitation, lower limb oedema, and no cardiac murmurs. Radiofemoral delay was not checked during initial assessment but was detected following the result of the CT scan. Initial investigations included an electrocardiography which showed sinus tachycardia with left ventricular hypertrophy. High sensitivity troponin-T was elevated at 55 ng/L (upper limit 14 ng/L), with no rise on serial testing. Chest X-ray revealed marked cardiomegaly with a cardiothoracic ratio of 0.68 and pulmonary congestion but there was no rib notching. TTE confirmed a severely dilated left ventricle (left ventricular internal dimension-diastole of 6.9 cm) with severe global systolic dysfunction, moderate concentric left ventricular hypertrophy, bicuspid aortic valve without significant gradient, and an estimated ejection fraction of 25–30%. Two weeks following admission he complained of sudden onset severe left arm pain and CT left upper limb confirmed embolic occlusion of the left brachial artery at the level of the distal third of the humerus and incidental coarctation of the aorta at the isthmus (Figure 1). The coarctation measures approximately 0.5–0.6 cm, compared with 2.0 cm in the distal arch and 2.7 cm in the proximal descending aorta. Follow-up TTE suggested the presence of coarctation of the aorta on a suprasternal view which was not performed at the time of his first TTE (Figure 2). The patient was then treated with intravenous diuretics along with other usual heart failure measures. The patient's heart failure responded very well to treatment. He lost 20 kilograms with diuretics and his blood pressure was adequately controlled. He also had successful embolectomy of his left brachial artery and was put on intravenous heparin and then had oral anticoagulation with warfarin. He is on regular follow-up in the cardiology clinic and is awaiting surgical intervention for his aortic coarctation.

## 3. Discussion

In this report, we presented a case of aortic coarctation that remained undiagnosed until the development of congestive cardiac failure in adulthood. Most cases of aortic coarctation are diagnosed in childhood due to the development of congestive cardiac failure or during adulthood due to treatment-refractory hypertension.

Dilated cardiomyopathy is a rare clinical presentation of coarctation of aorta in infants [1, 2]. One such case of dilated cardiomyopathy was reported by Ağaç et al. in 2012. The patient was a 55-year-old man who presented with symptoms of recent-onset cardiac failure [3]. The patient was successfully managed with stent implantation. Our patient, although stabilized at present, is still awaiting surgical intervention for correction of the coarctation at the time of preparation of the manuscript. More recently, another case of dilated cardiomyopathy secondary to aortic coarctation was reported; however, this patient had coarctation at an atypical location, namely, the aortic arch [4]. Our patient had the coarctation at the isthmus.

With regard to other symptoms at presentation, our patient also had newly diagnosed hypertension. However, during the initial evaluation his cardiac failure was attributed to the history of excessive alcohol consumption or possible myocarditis.

Taken together, our experience in this case highlights the need for the thorough evaluation of patients with heart failure and hypertension to rule out various causes of heart failure, including cardiomyopathy secondary to aortic coarctation. In conclusion, we presented an extremely rare case of dilated cardiomyopathy as a presentation of aortic coarctation in an adult patient. To the best of our knowledge, this appears to be only the third such case report in English literature.
